# Single-cell sequencing reveals heterogeneity between pancreatic adenosquamous carcinoma and pancreatic ductal adenocarcinoma with prognostic value

**DOI:** 10.3389/fimmu.2022.972298

**Published:** 2022-08-16

**Authors:** Deyu Zhang, Suna Wu, Shubo Pan, Meiqi Wang, Zhen Wang, Zixuan He, Guanghao Zhang, Fang Cui, Yihang Song, Wanshun Li, Xiaohua Shi, Haojie Huang, Huanhai Xu

**Affiliations:** ^1^ Department of Gastroenterology, First Affiliated Hospital, Naval Medical University, Shanghai, China; ^2^ Department of Gastroenterology , Yueqing People’s Hospital, Wenzhou, China; ^3^ Department of Gastroenterology , Affiliated Suzhou Science and Technology Town Hospital of Nanjing Medical University, Suzhou, China; ^4^ Academy of Medical Sciences, Zhengzhou University, Zhengzhou, China; ^5^ Department of Pancreatic Surgery, First Affiliated Hospital, Naval Medical University, Shanghai, China

**Keywords:** single cell sequencing, pancreatic adenosquamous carcinoma, pancreatic ductal adenocarcinoma, heterogeneity, prognostic value

## Abstract

Pancreatic adenosquamous carcinoma (ASPC) is a rare subtype of pancreatic cancer with lethal malignancy, and few studies have focused on the heterogeneity of ASPC. Here, we performed a single-cell sequencing procedure on pancreatic tumor tissue from an ASPC patient and a patient with high-grade intraductal papillary mucinous neoplasm (IPMN). Through the combined analysis of single-cell sequencing data from five pancreatic ductal adenocarcinoma (PDAC) patients, one IPMN patient, and one ASPC patient in a public database, we identified 11 main types of cells, including macrophages, B cells, cancer stem cells, ductal cells, fibroblasts, endo/stellate cells, neutrophils, acinar cells, T cells, natural killer (NK) cells, dendritic cells, and mast cells. Then, the different characteristics and differentiation paths of the immune microenvironment among IPMN, ASPC, and PDAC in macrophages, T cells, and cancer-associated fibroblasts (CAFs) were identified through multiple bioinformatics analyses. Two novel special cancer-associated fibroblasts were identified as nCAFs and imCAFs. Then, cancer cells in duct cells were identified using the infercnv software. Two ASPC-specific subgroups of cancer cells with squamous cell features were identified. Finally, the identified specific CAFs and cancer cells were mapped to TCGA-PAAD cohort through the cibersoftx software. All of these identified subgroups were calculated to have a significant prognostic value in pancreatic cancer patients. These findings will promote the clinical application of single-cell sequencing data of pancreatic cancer and deepen our understanding of ASPC.

## Introduction

Pancreatic cancer is one of the most malignant solid tumors. In 2018, it affected 450,000 people worldwide, and there were more than 40,000 related deaths. The overall 5-year survival rate of pancreatic cancer is less than 3.5%, indicating that it is a serious threat to the lives and health of patients worldwide (1). The major subtype of pancreatic cancer is pancreatic ductal adenocarcinoma (PDAC) (2). Pancreatic adenosquamous carcinoma (ASPC) is a rare subtype of malignant pancreatic tumor that accounts for 0.6%–4% of pancreatic exocrine tumors, with a reported incidence of 0.38%–10%. The mortality rate of pancreatic adenosquamous carcinoma is significantly higher than that of pancreatic ductal adenocarcinoma, and the prognosis of patients is extremely poor, with a median survival of only 4.4–13.1 months, and only a few patients survived for more than 1 year (3).

The histopathological characteristics of ASPC include the mixed presence of adenocarcinoma and squamous cell carcinoma tissues, and the squamous cell carcinoma component accounts for at least 30% of the total tissues (4). Squamous cell carcinoma tissues are mostly located in the center of the tumor and are prone to liquefaction necrosis in the early stage of ASPC. After collecting and analyzing 1,745 ASPC cases, Caitlin et al. reported that the proportion of squamous cell carcinoma in tumor tissue is not significantly correlated with the overall survival of patients (5). A series of studies illustrated that compared with PDAC, ASPC tumors are likely to have large diameters, occurring in the body part or tail part of the pancreas (4, 6). Lymph node or other organ metastasis and tumor embolus formation can occur at an early stage, along with liver metastasis and portal vein invasion.

Due to the low morbidity of ASPC, studies on this subtype are rare, and many aspects remain unclear. The most controversial aspect is the origin of ASPC. Pancreatic epithelial tissue does not contain a squamous component, and the origin of ASPC could be complicated. There are several hypotheses about the initial origin of ASPC. 1) A widely accepted hypothesis posits that after chronic inflammatory stimulation or biliary duct obstruction, pancreatic duct columnar epithelium cells undergo metaplasia to the squamous-like epithelium and then evolve into ASPC (7). 2) Tissue collision theory suggests that two histologically different tumor cells, i.e., columnar-like and squamous-like tumors, appear independently in the pancreas and peripheral tissue and subsequently form ASPC (8). 3) After carcinogen stimulation, pancreatic stem cells differentiate separately to form adenocarcinoma or squamous cell carcinoma, and then, these two components combine into ASPC (9). Studies focusing on the ASPC tumor microenvironment using single-cell sequencing analysis are also rare, and there is only one study related to ASPC. Xin et al. reported single-cell sequencing results from one ASPC sample, demonstrating that epidermal growth factor receptor (EGFR)-associated ligand–receptor pairs are activated in ductal-stromal cell communications (10). However, their study lacked a depiction of the ASPC tumor microenvironment and a comparison between PDAC and ASPC.

Moreover, intraductal papillary mucinous neoplasm (IPMN) is a papillary cystic tumor that originates from the main and or branch pancreatic ducts with the capability of secreting mucus. It has been recognized as a classical precancerous lesion in pancreatic cancer with a canceration rate of about 30% (11). Some studies illustrated that ASPC could originate from IPMN (12). However, the evolution path between IPMN and PDAC or ASPC is yet to be elucidated.

In the current study, single-cell sequencing was performed on pancreatic tumor tissue from an ASPC patient and a patient with high-grade intraductal papillary mucinous neoplasm. A combined analysis was conducted of single-cell sequencing data from five PDAC patients, one high-grade IPMN patient, and one ASPC patient, which were obtained from a public database. Eleven main types of cells, including macrophages, B cells, cancer stem cells, ductal cells, fibroblasts, endo/stellate cells, neutrophils, acinar cells, T cells, natural killer (NK) cells, dendritic cells (DCs), and mast cells, were identified. Then, the different characteristics and differentiation paths of the immune microenvironment among IPMN, ASPC, and PDAC in macrophages, T cells, and cancer-associated fibroblasts (CAFs) were identified through multiple bioinformatics analyses. Two novel special cancer-associated fibroblasts were identified as nCAFs and imCAFs. Then, cancer cells in duct cells were identified using infercnv software. Two ASPC-specific subgroups of cancer cells with squamous cell features were identified. Finally, the identified specific CAFs and cancer cells were mapped to TCGA-PAAD cohort through the cibersoftx software. All of these identified subgroups were calculated to have a significant prognostic value in pancreatic cancer patients. These findings will promote the clinical application of single-cell sequencing data of pancreatic cancer and deepen our understanding of ASPC.

## Materials and methods

### Patients and involved samples

Between January 2020 and March 2021, one ASPC pancreatic sample and one IPMN pancreatic sample were harvested in Changhai Hospital, Shanghai, during Whipple surgery. The diagnosis of IPMN and ASPC was made according to the intraoperative pathological diagnosis. Written informed consent was acquired from all patients. The Ethics Committee of Changhai Hospital, Shanghai, approved the current study. Another two pancreatic cancer cohorts were acquired from the Gene Expression Omnibus (GEO) database (https://www.ncbi.nlm.nih.gov/geo/), including GSE155698 and GSE165399. First, five PDAC samples (PDAC1–PDAC5) from GSE155698 were acquired (13). Then, one ASPC sample and one IPMN sample were acquired from GSE165399 (10).

### Single-cell sequencing procedure

Chromium Single Cell 3′ Reagent v3 kits were used to prepare libraries according to the manufacturer’s protocol. Single-cell suspensions were loaded onto the Chromium Single Cell Controller Instrument (10x Genomics, Pleasanton, CA, USA) to generate single-cell gel beads in emulsions (GEMs). After the generation of GEMs, reverse transcription reactions were performed. Then, cDNA was amplified, fragmented, end-repaired, A-tailed, index adapter ligated, and subjected to library amplification. Every library was sequenced on a NovaSeq 6000 platform (Illumina, San Diego, CA, USA), and 150-bp paired-end reads were generated. The Cell Ranger software pipeline (version 3.1.0) provided by 10x Genomics was used to demultiplex cellular barcodes, map reads to the genome and transcriptome using the STAR aligner, and downsample reads as required to generate normalized aggregate data across samples, producing a matrix of gene counts versus cells.

### Quality control and data correction

Quality control and data correction for single-cell samples were based on the number of detected genes, the number of detected molecules, and the percentage of mitochondrial, ribosomal, and hemoglobin genes from each single-cell sample. In detail, for all of the datasets, including our local datasets GSE155698 and GSE165399, samples with fewer than 1,000 genes, more than 3,000 genes, fewer than 200 molecules, more than 1% mitochondrial genes, and more than 2% ribosomal genes were removed. The remaining data in the three datasets were later used to produce a combined dataset.

### Removal of batch effect, integration, dimensionality reduction, clustering and visualization, and cluster annotation

After quality control, Seurat R package v4.0.2 was used to process the data (14). Harmony, an integration algorithm, was used to integrate the abovementioned three datasets and perform dimensionality reduction (15). Then, the NormalizeData() function was used to normalize the count data in the RNA assay by the LogNormalize method. With the help of the sharing nearest neighbor (SNN) modularity optimization-based clustering algorithm and Uniform Manifold Approximation and Projection (UMAP) algorithm, all cells were expressed in two-dimensional coordinates for visualization.

### Calculation and display of differentially expressed genes

The FindAllMarkers() and FindMarkers() functions of the scran package were used to perform the Wilcoxon test between pairs of cell clusters to find the genes specifically expressed in each cluster. According to the calculation results, the ggplot2 and heatmap packages were used to visually display the heat, violin, and bubble maps.

### Identification of significantly related pathways in different neutrophil cell types

To assess whether the gene set is enriched in a neutrophil cell subpopulation, the ‘irGSEA’ package (https://github.com/chuiqin/irGSEA/) in R software was used. This package was used to score individual cells using multiple gene set enrichment methods and generate a multiple gene set enrichment score matrix. Then, the Wilcoxon test was used to calculate the differentially expressed gene sets of each cell subpopulation in the enrichment fraction matrix of each gene set. Some specific enriched pathways were marked and visualized in single plots.

### Pseudotime analysis

Monocle2 (http://cole-trapnell-lab.github.io/monocle-release) was used to execute the single-cell trajectory analysis utilizing DDR-Tree and default parameters. Marker genes of the Seurat (version 4.0.2) clustering result and raw expression counts of the cell passed filtering were selected. On the basis of pseudotemporal analysis, the branch expression analysis model (BEAM Analysis) was used to analyze branch fate-determining genes.

### Analysis of cell differentiation trajectory

Monocle2 was used to order cells along the trajectories based on the pseudotime in the mesenchyme cells. The expression matrix of the mesenchymal cells derived from the Seurat object was submitted to Monocle3. The new_cell_data_set() function was used to create a cds object and perform dimensionality reduction, cell clustering, and differentiation trajectory inference.

### Chromosome copy number variation analysis

The inferCNV (V1.6.0) method with recommended parameters for 10x data was used to illustrate the diverse patterns of chromosome copy number variation in tumor cell clusters. The macrophage cells were used as the reference.

### The cancer genome atlas pancreatic cancer data acquisition

Pancreatic cancer sequencing data from The Cancer Genome Atlas (TCGA-PAAD) database were screened. The standardized RNA-sequence counts and clinical files were downloaded from TCGA data portal on 18 March 2022. A total of 180 samples with complete clinical follow-up information were obtained.

### Subtypes from single-cell sequencing estimation in TCGA-PAAD bulk sequencing data and Kaplan–Meier survival curve analysis

The downloaded TCGA data and subtype matrix acquired from Seurat analysis were uploaded to cibersoftx (https://cibersortx.stanford.edu/runcibersortx.php). The relative enrichment score of target subtypes in TCGA data was acquired through cibersoftx deconvolution analysis. The enrichment score of each sample in TCGA-PAAD was combined with their prognostic data (survival times). For the integrated dataset, Kaplan–Meier survival curves of different subtype gene sets in the dataset were drawn with the best cutoff using the Survival package. The OS rate from diagnosis to death or the last follow-up was calculated.

## Results

### Cell clustering of the landscape combined with intraductal papillary mucinous neoplasm, pancreatic ductal adenocarcinoma, and pancreatic adenosquamous carcinoma

**Figure 1:**
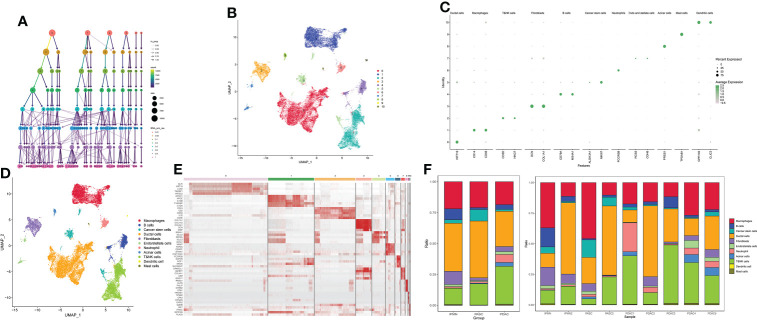
**(A)** Cluster tree of subgroup amounts at different resolution ratios. **(B)** UMAP cluster plot shows the divided subgroup before annotation. **(C)** Annotation of each subgroup with markers. **(D)** UMAP cluster plot shows the divided subgroup after annotation. **(E)** Heatmap shows the significantly expressed genes in each cluster. **(F)** Bar graph of the proportion of each identified subgroup in each sample or group. UMAP, Uniform Manifold Approximation and Projection.

After quality filtration, 45,238 cells were obtained for subsequent analysis; 0.05 was chosen to display the subgroup in the initial analysis (**Figures 1A, B**). The cells were catalogued into distinct cell lineages annotated with canonical marker gene expression (**Figure 1C**). As a result, macrophages, T$NK cells, B cells, cancer stem cells, ductal cells, fibroblasts, endo/stellate cells, neutrophils, acinar cells, dendritic cells, and mast cells were identified (**Figure 1D**). The highly expressed genes in each cluster are shown in **Figure 1E**. In summary, there are a greatly increased proportion of cancer stem cells and duct cells in ASPC tissue, indicating that the phenotype of ASPC cancer cells is more malignant than that of PDAC cells. The proportion of B cells, NK cells, and T cells in ASPC tissue was significantly less than that in PDAC tissue, revealing that the infiltration of immune cells could be difficult in ASPC (**Figure 1F**).

### M2-like macrophages tend to progress in the tumor microenvironment of pancreatic adenosquamous carcinoma

**Figure 2:**
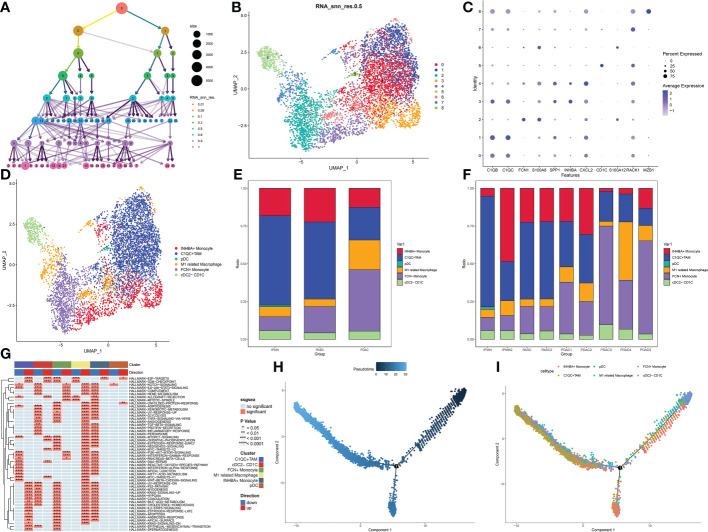
**(A)** Cluster tree of subgroup amounts at different resolution ratios. **(B)** UMAP cluster plot shows the divided subgroup before annotation. **(C)** Expression of each marker in each subgroup. **(D)** UMAP cluster plot shows the divided subgroup after annotation. **(E)** Bar graph of the proportion of each identified subgroup in each sample. **(F)** Bar graph of the proportion of each identified subgroup in each group. **(G)** GSVA plot showing the differentially enriched pathways in each identified subgroup. **(H, I)** Pseudotime analysis of these annotated groups. UMAP, Uniform Manifold Approximation and Projection; GSVA, gene set variation analysis.

The cluster tree plot shows different resolution ratios, and 0.5 was chosen for subsequent analysis (**Figures 2A, B**). To make the correct annotation, the marker genes of the macrophage subgroup were based on a previous study on the definition of macrophage subtypes from Zhang et al. (16). C1QB and C1QC were used to identify C1QC+ TAMs. SPP1, CXCL2, and INHBA were used to identify INHBA+ monocytes. According to Zhang’s study, these two kinds of macrophages are defined as M2 macrophages and are related to immune inactivation in the tumor microenvironment. FCN1 and S100A8 were used to identify FCN+ monocytes. This kind of macrophage represents an initial stage of macrophage chemotaxis from peripheral blood to the pancreatic tumor region. S100A8 and S100A12 were used to identify a subgroup related to M1 macrophages with antitumor activation. CD1C was used to identify conventional DCs (cDCs), and RACK1 and MAZB1 were used to identify plasmacytoid DCs (pDCs) (**Figures 2C, D**). As shown in the bar plot, M1-like macrophages show a significant decrease in ASPC compared to PDAC. Additionally, M2-related macrophages, including INHBA+ monocytes and C1QC+ tumor-associated macrophages, were significantly increased in ASPCs, followed by a reduction in juvenile macrophages in the tumor microenvironment (FCN+ monocytes) (**Figures 2E, F**). C1QC+ tumor-associated macrophages and INHBA+ monocytes showed a significant reduction in inflammation-related pathways, including the interferon alpha pathway, IL-6 pathway, and interferon gamma pathway (**Figure 2G**). Pseudotime analysis confirmed that FCN+ monocytes are the initial stage of all macrophages. Then, the monocytes could transfer to M1-like macrophages. Finally, during survival in the tumor microenvironment, macrophages tended to differentiate into two subtypes of M2 macrophages, INHBA+ monocytes and C1QC+ tumor-associated macrophages (**Figure 2H**).

### T cells in pancreatic adenosquamous carcinoma tissue show a widely inactivated phenotype compared to pancreatic ductal adenocarcinoma

**Figure 3:**
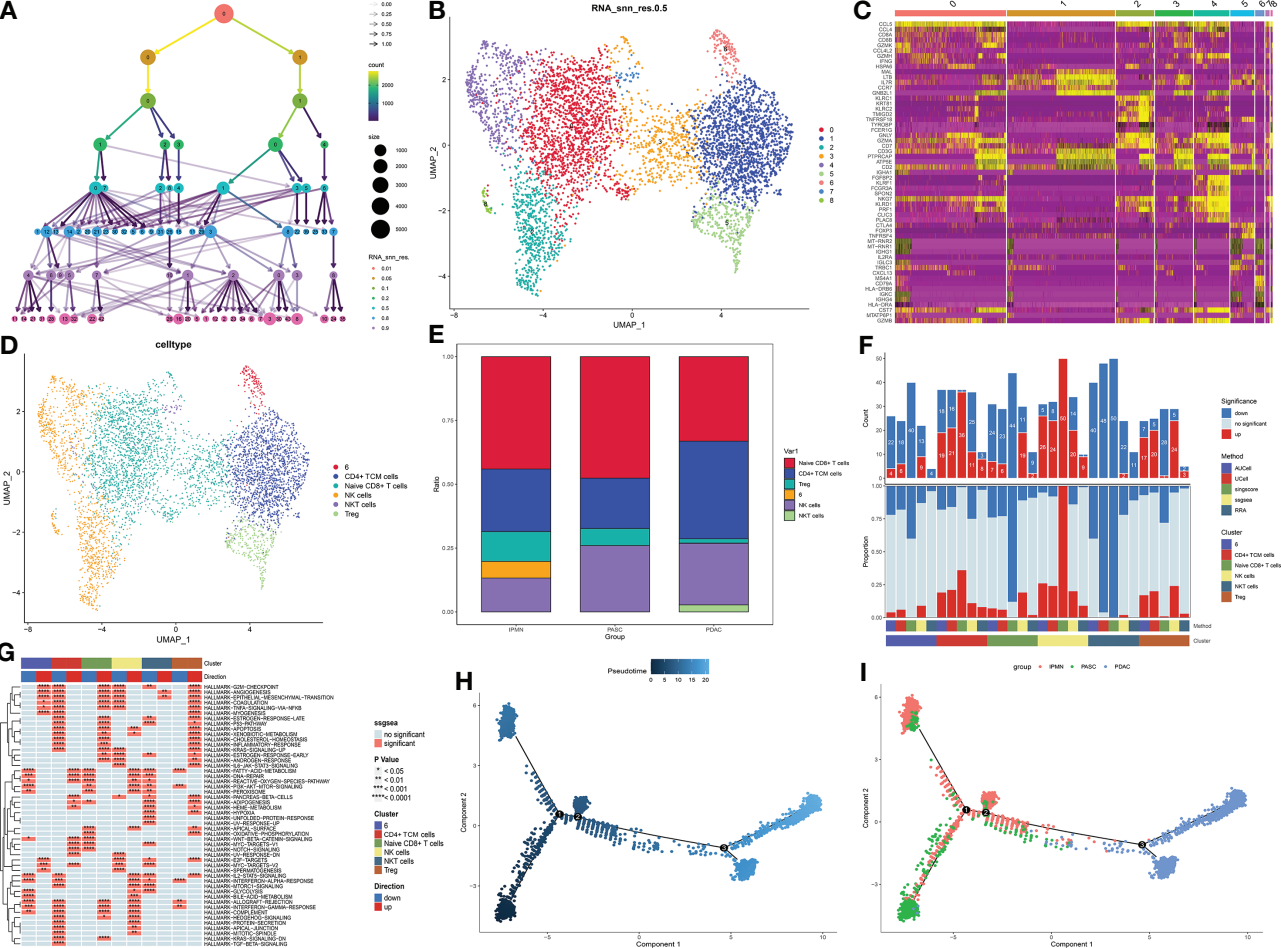
**(A)** Cluster tree of lymphocyte subgroup amounts at different resolution ratios. **(B)** UMAP cluster plot shows the divided subgroup before annotation. **(C)** Heatmap shows the significantly expressed genes in each cluster. **(D)** UMAP cluster plot shows the divided subgroup after annotation. **(E)** Bar graph of the proportion of each identified subgroup in each group. **(F)** Histogram showing the number of different enriched pathways in each identified subgroup. **(G)** GSVA plot showing the terms of different enriched pathways in each identified subgroup. **(H, I)** Pseudotime analysis of these annotated groups in ASPC, IPMN, and PDAC. UMAP, Uniform Manifold Approximation and Projection; GSVA, gene set variation analysis; ASPC, pancreatic adenosquamous carcinoma; IPMN, intraductal papillary mucinous neoplasm; PDAC, pancreatic ductal adenocarcinoma.

The cluster tree plot shows different resolution ratios, and 0.2 was chosen for subsequent analysis (**Figures 3A, B**). As shown in **Figure S1** and **Figures 2C, D**, groups 0 and 3 were identified as naïve CD8+ T cells (XCL1 and XCL2). Group 1 was identified as CD4+ central memory T cells (CD4+ Tcm, CCR7 CD40LG). Groups 2 and 4 were identified as NK (natural killer) cells (NKG7). Group 5 was identified as Treg (Foxp3). Group 7 was identified as NKT cells because of the double-positive expression of CD3 and NKG7. However, group 6 could not be defined, and the reason may be the sequencing error and unidentified double cells. The proportion of naïve CD8+ T cells showed a great increase in ASPC tissue, and CD4+ TCM cells were increased in PDAC tissue, followed by an ascending proportion of NKT cells (**Figure 3E**). Gene set variation analysis (GSVA) shows that NK cells and CD4+ CTM cells have a wide activation of pathways. In contrast, naïve CD8+ T cells were widely inactive (**Figure 3F**). Interestingly, CD8+ naïve T cells were enriched in the epithelial–mesenchymal transition pathway, indicating that CD8+ naïve T cells could promote a malignant phenotype in pancreatic cancer (**Figure 3G**). Pseudotime analysis indicated that the transition between IPMN and ASPC in T cells and NK cells could be minor, and the T cells and NK cells in PDAC could be different from ASPC and IPMN.

### Two novel subtypes of cancer-associated fibroblasts are identified with inactivation or full activation phenotype

**Figure 4:**
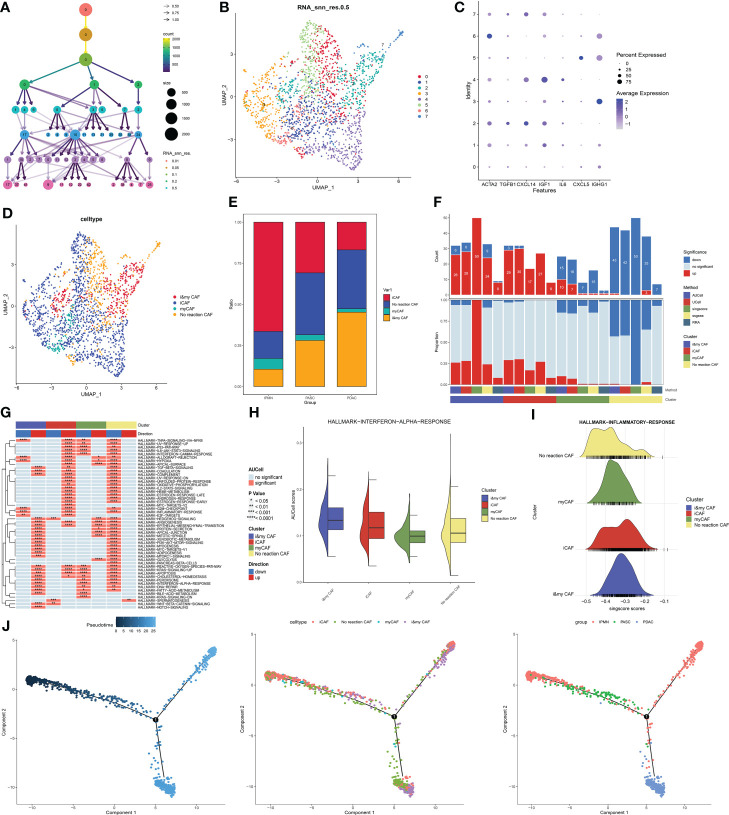
**(A)** Cluster tree of lymphocyte subgroup amounts at different resolution ratios. **(B)** UMAP cluster plot shows the divided subgroup before annotation. **(C)** Expression of each marker in each subgroup. **(D)** UMAP cluster plot shows the divided subgroup after annotation. **(E)** Bar graph of the proportion of each identified subgroup in each group. **(F)** Histogram showing the number of different enriched pathways in each identified subgroup. **(G)** GSVA plot showing the terms of different enriched pathways in each identified subgroup. **(H)** Gene set score of the interferon-α pathway. **(I)** Gene set score of the response to the inflammatory pathway. **(J)** Pseudotime analysis of these annotated groups in ASPC, IPMN, and PDAC. UMAP, Uniform Manifold Approximation and Projection; GSVA, gene set variation analysis; ASPC, pancreatic adenosquamous carcinoma; IPMN, intraductal papillary mucinous neoplasm; PDAC, pancreatic ductal adenocarcinoma.

The cluster tree plot shows different resolution ratios, and 0.5 was chosen for subsequent analysis (**Figures 3A, B**). The whole CAF cells were divided into eight groups. According to the classical definition of CAF subtypes (17), the main subtypes of CAFs are myofibroblastic CAFs (myCAFs) and inflammatory CAFs (iCAFs). MyCAFs mainly perform fibrogenesis, and ATCA2 and TGFB1 are the marker genes of myCAFs. ICAFs have more competence to react to inflammatory responses and produce a large series of inflammatory cytokines. The marker genes of iCAFs that were selected in this study were CXCL14, IGF1, IL6, CXCL5, and IGHG1. As shown in **Figure 4C**, according to the expression of myCAFs and iCAFs, four CAF subtypes were identified, including two common CAF and two novel CAF subtypes. For two common CAF subtypes, groups 1, 3, 4, and 5 were iCAFs, and group 6 was a myCAF. For two novel CAF subtypes, group 2 was identified as imCAFs because gene markers from both iCAFs and myCAFs were activated, and groups 0 and 7 were identified as nCAFs because gene markers from both iCAFs and myCAFs were inactivated (**Figures 4C, D**). Then, a significant increase in imCAFs in PDAC compared to ASPC was observed (**Figure 4E**). Additionally, in line with the definition of each kind of CAF, the amount of activated pathways in GSVA showed an ascendant tendency from non-reactive CAFs to imCAFs (**Figure 4F**). Moreover, nCAFs showed a wide range of downregulated inflammatory pathways, and imCAFs were active in multiple inflammatory pathways (**Figures 4G-I**). These results reveal different characteristics of CAF subtypes, and nCAF could be insensitive to chemotherapy and targeted therapy. Finally, pseudotime analysis revealed the evolutionary characteristics of CAFs (**Figure 4J**). ICAFs could be the initial phenotype of CAFs, and the microenvironment of PDAC is enriched with non-reactive CAFs compared with ASPCs.

### Cancer cells have different characteristics between pancreatic adenosquamous carcinoma and pancreatic ductal adenocarcinoma

**Figure 5:**
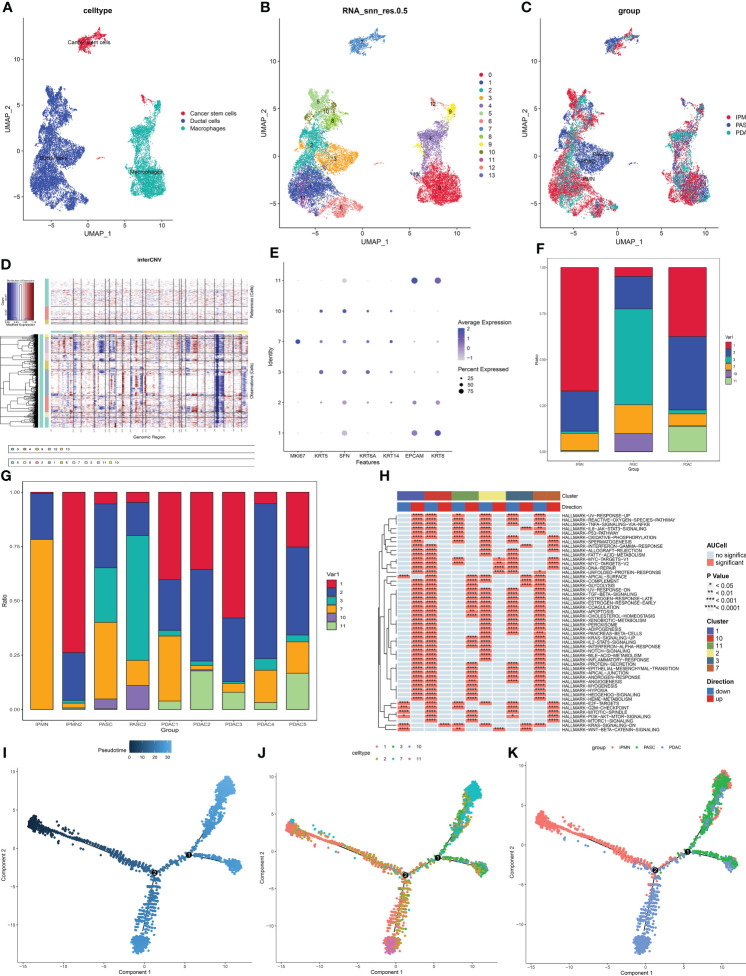
**(A)** UMAP plot of cancer stem cells, ductal cells, and macrophages. **(B)** UMAP plot of subgroups in cancer stem cells, ductal cells, and macrophages. **(C)** UMAP plot of cancer stem cells, ductal cells, and macrophages among ASPC, IPMN, and PDAC. **(D)** Infercnv plot identified malignant cells in duct cells and cancer stem cells. **(E)** Expression of squamous and columnar epithelium markers in the subgroups. **(F)** Bar graph of the proportion of each identified subgroup in each group. **(G,H)** Bar graph of the proportion of each identified subgroup in each sample. **(I–K)** Pseudotime analysis of these annotated groups in ASPC, IPMN, and PDAC. UMAP, Uniform Manifold Approximation and Projection; ASPC, pancreatic adenosquamous carcinoma; IPMN, intraductal papillary mucinous neoplasm; PDAC, pancreatic ductal adenocarcinoma.

Then, the heterogeneity of cancer cells among IPMN, ASPC, and PDAC was identified and portrayed. To identify and confirm cancer cells in duct cells and cancer stem cells, the infercnv procedure was performed. Macrophages were chosen as the normal cell control, and these three types of cells were divided into 14 groups (**Figures 5A, B**). The UMAP plot shows the different distributions between PDAC and ASPC (**Figure 5C**). According to the infercnv plot, subgroups 5, 6, and 8 were identified as normal ductal cells and were excluded from our subsequent studies. Then, by staining for squamous epithelium markers (including KRT5, KRT6A, SFN, and KRT14) and columnar epithelium markers (including EPCAM and KRT8), groups 3, 7, and 10 were identified as squamous cancer cells, and groups 5, 6, and 8 were identified as adenocarcinoma cells (**Figures 5D, E**). The different proportions of subgroups among IPMN, PDAC, and ASPC confirmed that groups 3, 7, and 10 were significantly enriched in the ASPC group and ASPC samples (**Figures 5F, G**).

According to the GSVA results, proliferation-related pathways, including the G2M checkpoint pathway and mitotic pathway, were enriched in group 7, indicating that group 7 could be the promoter of ASPC carcinogenesis. Cancer-related pathways, including the epithelial–mesenchymal transition pathway and angiogenesis, were enriched in group 1, indicating that group 1 plays a pivotal role in PDAC development (**Figure 5H**). Pseudotime analysis shows that the differentiation paths in PDAC and ASPC are different. Both of them could develop from IPMN, and then the paths of ASPC and PDAC are divided. Finally, ASPC could be divided into two subtypes. One subtype contained less adenocarcinoma than the other (**Figures 5I-K**).

### The identified subgroup of PACS in cancer cells and cancer-associated fibroblasts has great prognostic value in pancreatic cancer patients

**Figure 6:**
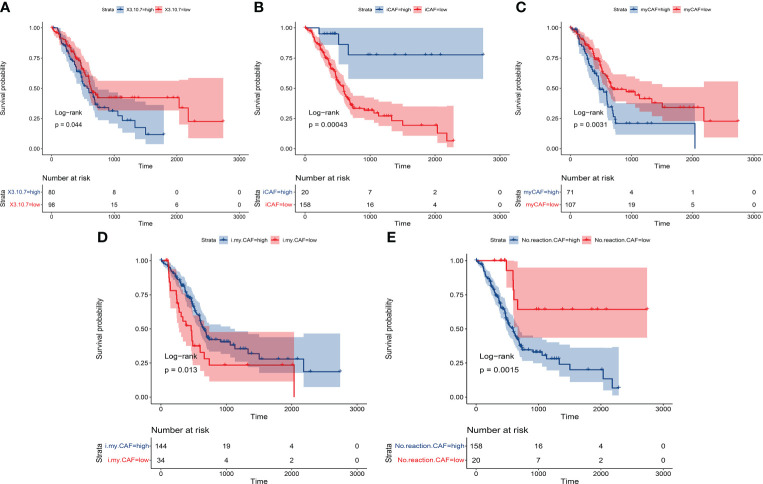
The Kaplan–Meier plot shows that the previously identified subgroups had significant prognostic value in patients. **(A)** ASPC-specific cancer cell subtypes (combined expression of groups 3, 7, and 10). **(B)** Identified iCAF subgroup. **(C)** Identified myCAF subgroup. **(D)** Identified imCAF subgroup. **(E)** Identified nCAF subgroup. ASPC, pancreatic adenosquamous carcinoma; iCAF, inflammatory cancer-associated fibroblast; myCAF, myofibroblastic cancer-associated fibroblast.

Finally, the prognostic value of our identified subgroup in cancer cells and CAFs was explored. As described in the Materials and Methods section, the seurat matrix of each subgroup was extracted and uploaded to the cibersoftx software. Then, the count matrix of bulk RNA sequencing data acquired from TCGA-PAAD was also uploaded to the cibersoftx software. The estimated proportion of each subgroup was calculated in TCGA data by a deconvolution algorithm. Combined with survival outcomes, high expression of ASPC-specific cancer cell subtypes (combined expression of groups 3, 7, and 10) or identified nCAF subgroup in pancreatic cancer patients was correlated with poor clinical outcomes (**Figures 6A, E**). High expression of the identified iCAF subgroup, myCAF subgroup, or imCAF subgroup was associated with favorable clinical outcomes (**Figures 6B-D**).

## Discussion

As described in the Introduction, pancreatic cancer is a common kind of malignant tumor with high morbidity and mortality worldwide. The main kind of pancreatic cancer is PDAC, which accounts for nearly 80% of the morbidity of pancreatic cancer (1). Additionally, pancreatic cystic tumors are considered to be the precursors of pancreatic cancer, among which the most common is IPMN. Most IPMNs are low-grade heteroplastic hyperplasia, but some IPMNs are malignant and can develop into pancreatic adenocarcinoma with a worse prognosis (18). Bernard et al. (19) performed single-cell RNA sequencing on 5,403 cells from two low-grade IPMN, two high-grade IPMN, and two pancreatic cancer specimens and analyzed the heterogeneity changes in epithelial cells and the tumor microenvironment during cancer development. They reported that both oncogenic gene expression and tumor suppressor gene expression were unregulated in low-grade IPMN. However, the expression of these tumor suppressor genes disappeared in high-grade IPMN and PDAC. Additionally, they also reported that during the process of high-grade IPMN progression to PDAC, iCAFs are upregulated and often accompanied by the formation of an immune escape microenvironment. Some studies have focused on the investigation of heterogeneous PDAC compared to normal pancreatic tissue. Peng et al. used single-cell transcriptome sequencing to explore the internal heterogeneity of pancreatic cancer and regulators in the progression of PDAC. This study reports two types of ductal cell subsets with different malignant gene expression profiles, including the ductal cell subsets with unique proliferative characteristics. In addition, this study demonstrated the role of abnormal pathways such as Wnt and Notch in pancreatic cancer and identified new genes such as EGLN3, MMP9, and FOS KLF5 and other transcription factors involved in carcinogenesis (20). In our current research, some new phenotypes and characteristics were identified between high-grade IPMN and PDAC. PDAC tissues exhibit an inflammatory phenotype in the tumor microenvironment, especially in macrophages, and the epithelial–mesenchymal transition pathway is enriched in PDAC-related cancer cells.

The most interesting finding in our current analysis is the exploration of heterogeneity between ASPC and PDAC. In fact, our current study is not the first to focus on the single-cell pattern of ASPC. Xin et al. reported single-cell sequencing results from one ASPC sample, demonstrating that EGFR-associated ligand–receptor pairs are activated in ductal-stromal cell communications (10). However, their study has some questions that need to be explored. First, their study lacked a depiction of the ASPC tumor microenvironment, and the sample size was too short in this study to draw a concrete conclusion. Second, as described in the Introduction, the origin of ASPC has yet to be fully clarified, and some studies have illustrated the potential correlation between PDAC and ASPC (7, 8). Based on differential transcription factor expression of samples with >40% cellularity from resectable primary pancreatic cancer, Bailey et al. described four subtypes using machine learning methods, including squamous, pancreatic progenitor, immunogenic, and aberrantly differentiated endocrine exocrine. Among them, the squamous subtype has notable pan-squamous features, including a significant association with ASPC histology (21). These findings illustrated that ASPC has a potential relationship with squamous-like PDAC. However, both ASPC and PDAC samples were not included in their study simultaneously. In our study, we performed single-cell sequencing in one ASPC tissue and one high-grade IPMN tissue. Then, we involved the ASPC and IPMN samples from the study of Xin et al. (10) and five PDAC samples from the study of Steele et al. (13). The immune-related microenvironment is different between PDAC and ASPC. Specifically, M1-like macrophages show a significant decrease in ASPC compared to PDAC. M2-related macrophages, including INHBA+ monocytes and C1QC+ tumor-associated macrophages, were significantly increased in ASPC, following a reduction in juvenile macrophages in the tumor microenvironment (FCN+ monocytes). However, the proportion of naïve CD8+ T cells shows a great increase in ASPC tissue, and CD4+ TCM cells are increased in PDAC tissue, followed by an ascending proportion of NKT cells. These results indicate the complex heterogeneity of the immune-related microenvironment in ASPC and PDAC.

Another interesting finding in our research is that we identified two novel subtypes of CAFs. According to the classical definition of CAF subtypes (17), the main subtypes of CAFs are myCAFs and iCAFs. MyCAFs mainly perform fibrogenesis. ICAFs have more competence to react to inflammatory responses and produce a large series of inflammatory cytokines. However, in our current study, we identified two novel CAFs. One subtype is named ‘imCAF’ because this subgroup expresses both fibrogenesis-related genes and genes related to inflammatory cytokines. Another subtype is named ‘nCAF’ because this subgroup expressed neither fibrogenesis-related genes nor genes related to inflammatory cytokines. Then, we observed a significant increase in imCAFs in PDAC compared to ASPC, indicating that PDAC could have better chemoreactivity than ASPC.

Our study also analyzed the origin of ASPC at single-cell resolution. There are several hypotheses about the initial origin of ASPC: 1) after chronic inflammatory stimulation or biliary duct obstruction, pancreatic duct columnar epithelium cells undergo metaplasia to the squamous-like epithelium and then evolve into ASPC, and this hypothesis is widely accepted by scholars worldwide (7). 2) Tissue collision theory: two histologically different tumor cells, columnar-like and squamous-like tumors, appear independently in the pancreas and peripheral tissue and subsequently form ASPC (8). 3) After carcinogen stimulation, pancreatic stem cells differentiate separately to form adenocarcinoma or squamous cell carcinoma, and then, these two components combine into ASPC (9). By comparing the potential differentiation path between IPMN to PDAC and IPMN to ASPC, our analysis strongly supported the first hypothesis because we observed an early separation of the differentiation path between IPMN to PDAC and IPMN to ASPC. This finding could deepen our understanding of carcinogenesis in ASPC.

However, this study also has some limitations. Firstly, the results in this study are based on bioinformatics analysis of our clinical samples without immunofluorescence validation. Further validation in immunofluorescence could strengthen our findings. Secondly, the number of enrolled samples is small. More samples are needed for further research.

## Conclusion

In conclusion, we examined the microenvironmental changes among IPMN, PDAC, and ASPC during ASPC progression from a single-cell perspective.

Two novel special cancer-associated fibroblasts were identified as nCAFs and imCAFs. Then, two ASPC-specific subgroups of cancer cells with squamous cell features were identified. Finally, the identified specific CAFs and cancer cells were mapped to TCGA-PAAD cohort through the cibersoftx software. All of these identified subgroups were calculated to have a significant prognostic value in pancreatic cancer patients. These findings provide valuable information to understand the critical microenvironment underlying PDAC and ASPC and demonstrate potential therapeutic targets for pancreatic cancer.

## Data availability statement

The datasets presented in this study can be found in online repositories. The names of the repository/repositories and accession number(s) can be found below: https://ngdc.cncb.ac.cn/, PRJCA010063.

## Ethics statement

This study was reviewed and approved by the Ethics Committee of First affiliated Hospital, Naval medical University. The patients/participants provided their written informed consent to participate in this study.

## Author contributions

HX, HH and XS designed the whole study and provided financial support. DZ, SW, SP, MW, ZW, ZH, GZ and FC collected tumor tissue and developed the codes, drew the plots. DZ, YS, and WL wrote the original draft. All authors contributed to the article and approved the submitted version.

## Acknowledgments

We thank OE Biotech Co., Ltd. (Shanghai, China), for providing single-cell RNA-seq and Dr. Yongbing Ba for assistance with bioinformatics analysis.

## Conflict of interest

The authors declare that the research was conducted in the absence of any commercial or financial relationships that could be construed as a potential conflict of interest.

## Publisher’s note

All claims expressed in this article are solely those of the authors and do not necessarily represent those of their affiliated organizations, or those of the publisher, the editors and the reviewers. Any product that may be evaluated in this article, or claim that may be made by its manufacturer, is not guaranteed or endorsed by the publisher.

## Supplementary material

Supplementary Figure 1 The expression of gene markers of each T-cell subtype.
