# Supplementary figures and images for: Single-cell sequencing reveals heterogeneity between pancreatic adenosquamous carcinoma and pancreatic ductal adenocarcinoma with prognostic value
Source: Front Immunol. 2022 Aug 16;13:972298. doi: 10.3389/fimmu.2022.972298 (PMC9424731; doi:10.3389/fimmu.2022.972298)

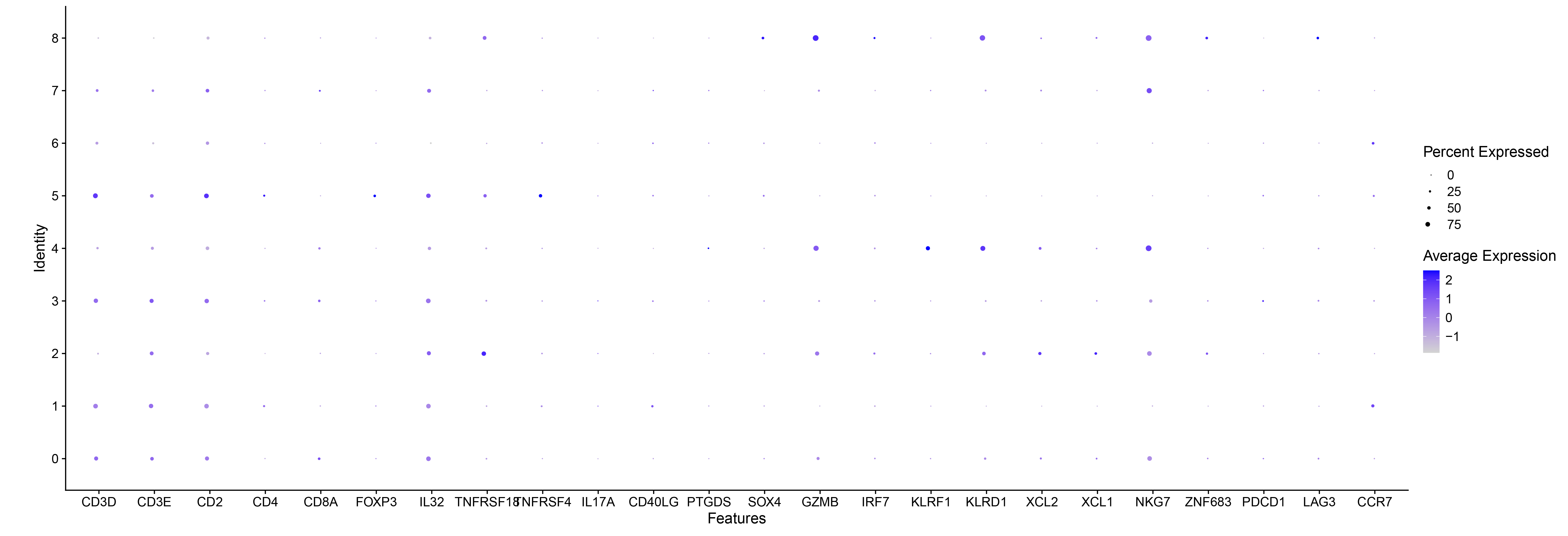

Supplement: Supplementary Figure 1 — The expression of gene markers of each T-cell subtype. [file Image_1.jpeg]
